# Haemoglobin, albumin, lymphocyte, and platelet score as an independent predictor for renal prognosis in IgA nephropathy

**DOI:** 10.3389/fendo.2024.1339921

**Published:** 2024-04-26

**Authors:** Yuan Yuan, Xiaoli Liang, Minhui He, Yufan Wu, Xue Jiang

**Affiliations:** ^1^ Department of Nephrology, Hangzhou TCM Hospital Affiliated to Zhejiang Chinese Medical University, Hangzhou, China; ^2^ Key Laboratory of Zhejiang Province, Management of Kidney Disease, Hangzhou, China; ^3^ Key Laboratory of Precise Prevention and Treatment of Rheumatism Syndrome of Renal Wind Disease, Hangzhou, China

**Keywords:** HALP, IgA nephropathy, prognosis, cohort study, clinicopathological correlation

## Abstract

**Objective:**

The haemoglobin, albumin, lymphocyte, and platelet (HALP) score, a convenient and composite laboratory biomarker, can reflect inflammation and systemic nutritional status. This study was performed to investigate the effect of the HALP score on the prognosis of patients with IgA nephropathy (IgAN).

**Methods:**

This is a retrospective single centre study that enrolled 895 biopsy-confirmed IgAN patients from June 2019 to June 2022 who were followed for more than 1 year. Kaplan–Meier curves and Cox regression analyses were performed to determine the relationship between HALP and adverse outcomes. The restricted cubic splines was used to identify the possible associations. The optimal cut-off value of HALP for renal poor outcome was identified by the area under the receiver operating characteristic curve (AUC).

**Results:**

A total of 895 patients finally participated in the study and were divided into three groups (tertial 1-3) according to the baseline HALP score. More severe clinicopathologic features were observed in the lower HALP group, and Kaplan−Meier analysis showed patients in tertial 1 had a higher risk of kidney failure than the other groups (log-rank=11.02, P= 0.004). Multivariate Cox regression revealed that HALP score was an independent risk factor for renal prognosis in IgAN (adjusted HR: 0.967, 95% CI: 0.945-0.990, P = 0.006). The results of subgroup analysis suggested that HALP was more important in patients under the age of 50, BMI ≤ 23.9 and eGFR ≤ 90 mL/min/1.73 m2. The best cut-off HALP for renal survival was 38.83, sensitivity 72.1%, and specificity 55.9% (AUC: 0.662). Patients were further grouped according to HALP cut-off values and propensity matched. Multivariate Cox regression analysis revealed that HALP remained an independent predictor of IgAN in the matched cohort (HR 0.222, CI: 0.084-0.588, P=0.002).

**Conclusion:**

HALP is a novel and potent composite parameter to predict kidney outcome in patients with IgAN.

## Introduction

IgA nephropathy (IgAN) is the most common primary glomerulonephritis among the world, and is characterised by the deposition of IgA1 in the mesangium of the glomeruli. Approximately 30-40% of patients gradually progress to end-stage renal disease (ESRD) within 20 years (1, 2). Previous studies have found that hypertension (3), proteinuria (4), impaired kidney function, persistent haematuria and hyperuricaemia (5, 6) are risk factors for IgAN progression. However, the underlying mechanisms of IgAN remain unknown. Therefore, early assessment of renal prognosis and take active intervention are crucial to decelerate progression to ESRD in IgAN. As IgAN is an immune-associated disease, a large number of researches have reported that IgAN is closely related to immunity and inflammation.

The haemoglobin, albumin, lymphocyte and platelet (HALP) scores, a combination of inflammatory and nutritional status, have been identified as an useful and widely prognostic indicators for various malignancies, such as renal cancer (7), lung adenosquamous carcinoma (8), gastrointestinal stromal tumour (9), and bladder cancer (10). Among haemodialysis and in the general population, the HALP score was independently associated with the risk of cardiovascular and all-cause mortality (11).

In recent years, HALP has emerged as a new prognostic biomarker in various malignant and acute diseases related to all-cause and cardiovascular mortality (12, 13), while the association of the HLPA score with renal prognosis in kidney disease, such as IgAN, is limited. Therefore, our study aimed to address this problem.

## Material and methods

**Figure 1:**
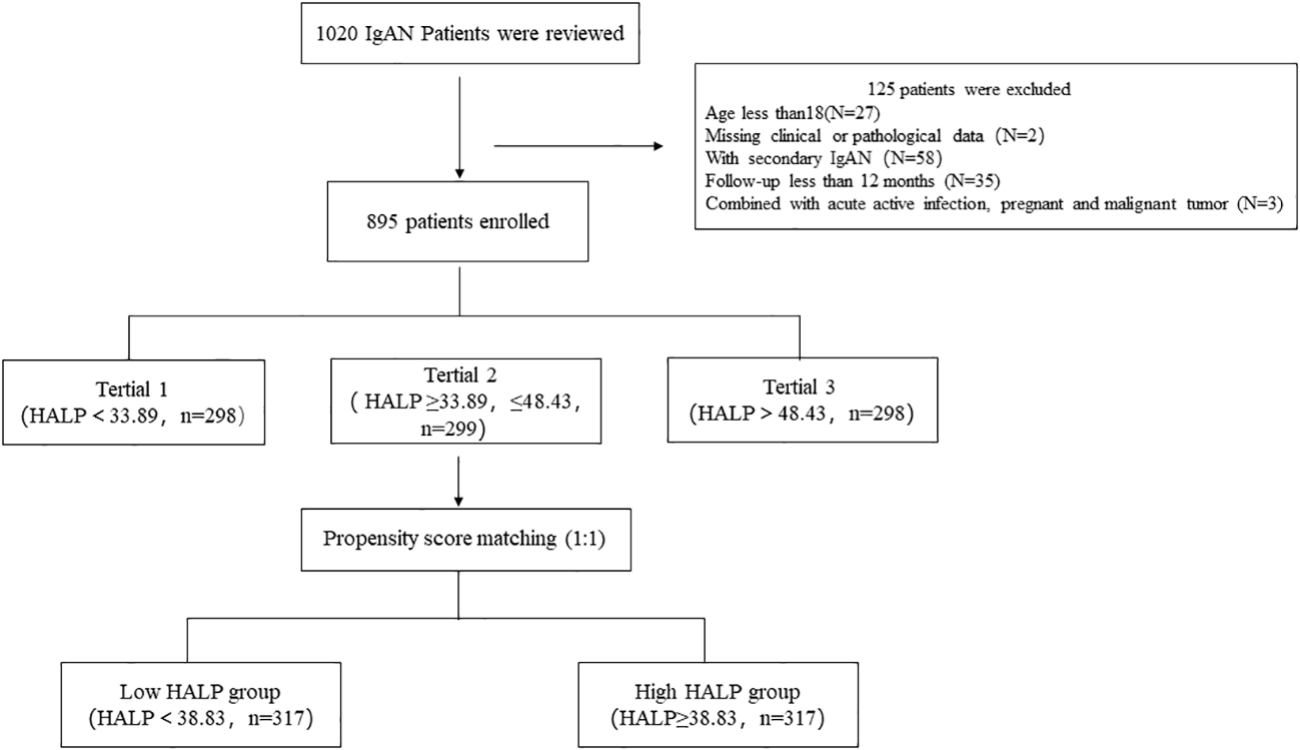
Flowchart showing the number of IgAN patients included in the analyses.

This single-centre retrospective study included 1,020 patients with IgAN diagnosed by renal biopsy at Hangzhou Hospital of Traditional Chinese Medicine between June 2019 and June 2022. Among them, 125 individuals were excluded for the following reasons: 27 patients less than 18 years old. Thirty-five individuals had missing data during follow-up or follow-up of less than 12 months, 3 patients had acute active infection, pregnancy and malignant tumours, and 58 patients had potential secondary causes of IgAN, such as liver diseases, autoimmune disorders and Henoch-Schönlein purpura. Two patients did not have sufficient clinical or pathologic data. All patients were followed up for at least 12 months (**Figure 1**). The study was approved by the Ethics Committee of Hangzhou Hospital of Traditional Chinese Medicine and is in accordance with the principles of the Declaration of Helsinki. (No. 2023KLL001).

### Clinical data

Medical records of each patient were carefully reviewed, and all data were collected at the time of renal biopsy, including demographics (age, sex, MAP, BIM, hypertension and diabetic history), clinical data (serum creatinine (Scr), uric acid (UA), haemoglobin (Hb), platelets (PLT), lymphocyte count, albumin, urine protein (U-pro), estimated glomerular filtration rate (eGFR)) and treatment plan.

### HALP definition

The blood count data collected at the time of biopsy were used to evaluate the markers needed for the study. The computational equation of HALP values was as follows: haemoglobin (g/L) × albumin (g/L) × lymphocytes (/L)/platelets (/L).

### Pathological data

Renal biopsy samples were evaluated by light microscopy, immunofluorescence and electron microscopy by two experienced pathologists and nephrologists according to the Oxford classification of IgAN: mesangial hypercellularity (M0/M1), endocapillary hypercellularity (E0/E1), segmental glomerulosclerosis (S0/S1), tubular atrophy/interstitial fibrosis (T0/T1/T2), and cellular or fibro-cellular crescents (C0/C1/C2).

### Prognosis definition

The primary endpoint event was defined as a composite event of either a 30% decline in the eGFR or ESRD (defined as eGFR <15 mL/min/1.73 m2 or need for kidney replacement therapy).

### Statistical analysis

All statistical analyses were performed using IBM SPSS software, version 19.0. Continuous distributions are displayed as the mean ± standard deviation, while skewed distributions are expressed as the median with interquartile range and were analysed by an unpaired t test, the Kruskal–Wallis H test or the nonparametric Mann–Whitney U test.

Categorical data are presented as frequencies and were analysed by χ2 or Fisher’s exact tests.

Survival curves of the kidney endpoint were analysed by the Kaplan−Meier method and the log-rank test. Cox regression were performed to analyse independent factors for IgAN prognosis. Besides, we implemented restricted cubic splines to study the potential nonlinear relationship between HALP continuous variables and the hazard renal endpoint. Subgroup analyses were performed by Cox regression models.

The cut-off and predictive values of HALP, platelet-to-lymphocyte ratio (PLR) and platelet-to-albumin ratio (PAR) for renal prognosis were assessed by receiver operating characteristic (ROC) curves.

A 1:1 propensity score (PS) match was carried out to eliminate important differences at baseline. According to the greedy matching algorithm, multilogit regression was performed to make all the parameters comparable at baseline. Kaplan−Meier survival analysis and Cox regression were then used on the new matched cohort. Significance was defined at a P value < 0.05.

## Results

### Baseline characteristics of all patients

A total of 1,020 IgAN patients were enrolled, while 125 individuals were excluded for the following reasons: secondary IgAN, combined with acute active infection, pregnancy and malignant tumour, follow-up less than 12 months or insufficient pathological data. Finally, a total of 895 patients were included in this study. This cohort study comprised 384 (42.9%) males and 511 (57.1%) females, and the mean age was 40.97 ± 12.261 years old. Based on the baseline HALP score, patients were divided into three groups. Tertial 1 (<33.89, n=298, account for 33.3%), Tertial 2 (≥33.89, ≤48.43, n=299, account for 33.4%), and Tertial 3 (>48.43, n=298, account for 33.3%). Analysis of baseline data of patients in each group showed that all clinical indices were significantly different between these groups, except BMI, MAP, prevalence of diabetes and hypertension. The MESTC score of renal pathology showed that the E, S, T, and C scores in all groups were significantly different. With the decrease in the HALP score, the proportion of patients with high scores (E1, S1, T1/2 and C1/2) gradually increased, and more patients received Steroid with/without Immunosuppressants treatment. which indicating that patients with low HALP scores had more serious renal pathological changes and need more aggressive treatment. As shown in **Table 1**.

**Table 1:** Baseline characteristics of different HALP score groups among study population.

Variable	Total (n=895)	Tertile 1 (n-298) <33.89	Tertile 2 (n=299) 33.89-48.43	Tertile 3 (n=298) >48.43	P value
Age, years	40.97±12.261	42.21±12.030	39.35±11.133	41.34±13.384	**0.009**
BMI, kg/m^2^	23.58±3.595	23.24±3.569	23.68±3.482	23.83±3.716	0.114
Male, n (%)	384 (42.9)	89 (29.9)	111 (37.1)	184 (61.7)	**0.000**
MAP, mmHg	96.13±14.055	96.67±14.524	95.88 (13.497)	95.85 (14.158)	0.724
Hypertension, n (%)	306 (34.2)	108 (36.2)	99 (33.1)	99 (33.2)	0.664
Diabetes, n (%)	27(3.4)	8 (2.7)	10 (3.3)	9 (3.0)	0.969
HB, g/L	127.13±19.241	115.2±17.04	126.86±15.8	139.34±16.764	**0.000**
PLT, 10^9^/L	227.81±60.232	254.85±65.323	226.4±51.426	202.19±51.045	**0.000**
Lymphocytes, 10^9^/L	1.96±0.574	1.62±0.451	1.91±0.444	2.34±0.569	**0.000**
Albumin, g/L	37.27±4.517	34.72±5.144	37.97±3.609	39.11±3.388	**0.000**
24-hour proteinuria, g/d	0.89 (0.46, 1.80)	1.25 (0.62, 2.69)	0.78 (0.42, 1.66)	0.75 (0.39, 1.40)	**0.000**
U-pro, n (%)					**0.000**
-	96 (10.7)	21 (7.0)	39 (13.0)	36 (12.1)	
+	352 (39.3)	100 (33.6)	112 (37.5)	140 (47.0)	
++	367 (41.0)	129 (43.3)	131 (43.8)	107 (35.9)	
+++	80 (8.9)	48 (16.1)	17 (5.7)	15 (5.0)	
Scr, umol/L	79 (62, 109)	83 (62, 135.8)	74 (59, 101)	81 (65, 104.3)	**0.000**
eGFR, ml/min/1.73m^2^	81.83±32.398	73.14±35.234	86.56±31.955	85.79±27.92	**0.000**
UA, μmol/L	377.7±109.22	374.59±108.06	365.39±110.066	393.15±108.044	**0.007**
Mesangial cellularity, n (%)					0.298
M0	5 (0.6)	3 (1.0)	2 (0.7)	0 (0)	
M1	890 (99.4)	295 (99.0)	297 (99.3)	298 (100)	
Endocapillary hypercellularity, n (%)					**0.000**
E0	555 (62.0)	156 (52.3)	190 (63.5)	209 (70.1)	
E1	340 (38.0)	142 (47.7)	109 (36.5)	89 (29.9)	
Segmental sclerosis, n (%)					0.071
S0	187 (20.9)	49 (16.4)	69 (23.1)	69 (23.2)	
S1	708 (79.1)	249 (83.6)	230 (76.9)	229 (76.8)	
Tubular atrophy/interstitial fibrosis, n (%)					**0.000**
T0	617 (68.9)	177 (59.4)	216 (72.2)	224 (75.2)	
T1	217 (24.2)	84 (28.2)	68 (22.7)	65 (21.8)	
T2	61 (6.8)	37 (12.4)	15 (5.0)	9 (3.0)	
Crescents, n (%)					**0.000**
C0	319 (35.6)	86 (28.9)	102 (34.1)	131 (44.0)	
C1	523 (58.4)	175 (58.7)	184 (61.5)	164 (55.0)	
C2	53 (5.9)	37 (12.4)	13 (4.3)	3 (1.0)	
Therapy, n (%)					**0.000**
None Steroid	292 (32.6)	63 (21.1)	105 (35.1)	124 (41.6)	
Steroid alone	191 (21.3)	75 (25.2)	57 (19.1)	59 (19.8)	
Steroid+Immunosuppressants	412 (46.0)	160 (53.7)	137 (45.8)	115 (38.6)	

BMI, Body mass index; MAP, mean arterial pressure; HB, hemoglobin; PLT, platelete; eGFR, estimated glomerular filtration rate; Scr, serum creatinine; UA, uric acid. Continuous variables are expressed as mean±standard deviation or as median (interquartile range). Categorical variables are expressed as frequency (%). Bold values was that the differences were significant.

### Associations of HALP score with risk of renal survival rate

**Figure 2:**
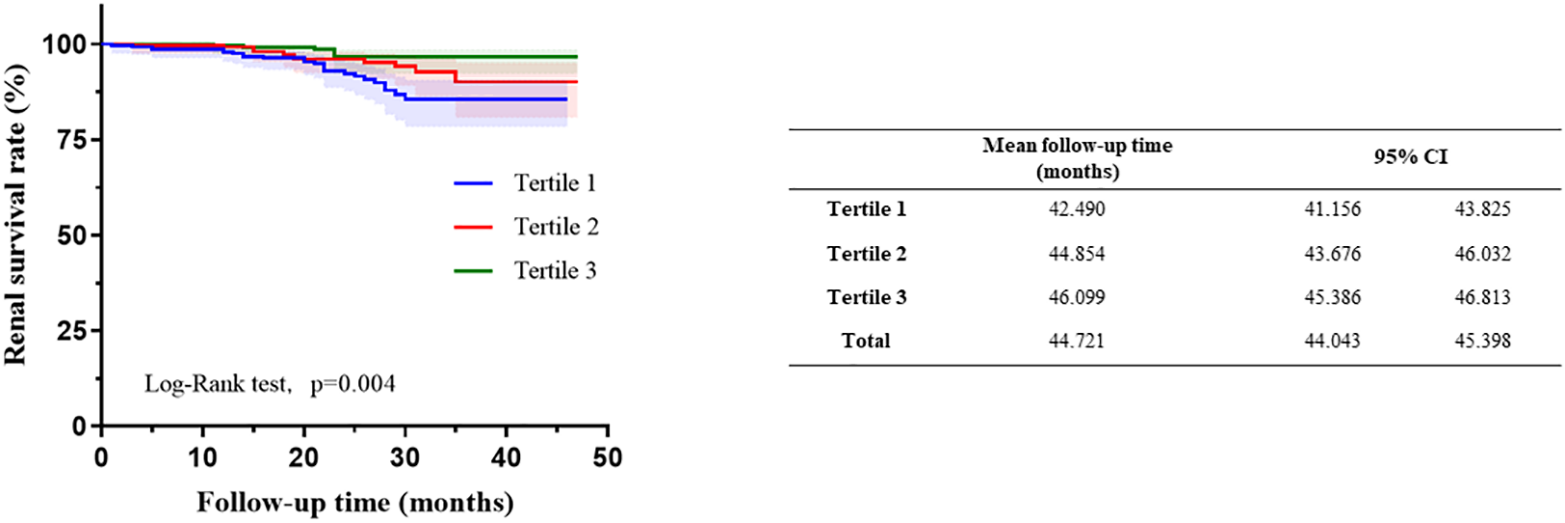
Kaplan–Meier curves of renal outcomes in different haemoglobin, albumin, lymphocyte, and platelet (HALP).

Among the 895 patients, the average follow-up time was 23.05 ± 8.91 months. There was no significant difference in the follow-up time among these groups. Renal endpoint event defined as a 30% decline in the eGFR or ESRD. The results showed that the incidence of endpoint events in the tertile1 group was 8.1%, while in the other groups, fewer people had endpoint events, accounting for 4.3% and 2.0%, respectively (P=0.002). Kaplan−Meier analysis indicated that participants in tertial 1 had a higher risk of developing kidney failure than the other groups (log-rank=11.02, P= 0.004). Patients in tertial 3 group had significantly higher mean kidney survival times (46.099 months, CI: 45.386-46.813) than those in the other two groups (**Figure 2**).

In unadjusted Cox analysis, Higher HALP score was connected with decreased exposure to poor renal prognosis in continuous variables [HR = 0.966, 95% CI: 0.946–0.987, P =0.001], while when HALP was treated as a categorical variable, the hazard of poor renal prognosis in Tertial 1 was higher than that in Tertial 2 (HR = 0.535, 95% CI: 0.272–1.051, P = 0.07) and Tertial 3 (HR = 0.259, 95% CI: 0.106–0.635, P = 0.003) (**Table 2**).

**Table 2:** Multivariate Cox regression analysis hemoglobin, albumin, lymphocyte, and platelet (HALP) and renal outcomes.

	Crude Model		Model 1		Model 2	
	HR(95%CI)	P value	HR(95%CI)	P value	HR(95%CI)	P value
Continuous	0.966(0.946-0.987)	**0.001**	0.965(0.943-0.988)	**0.003**	0.967(0.945-0.990)	**0.006**
Categories						
Tertile 1	1		1		1	
Tertile 2	0.535(0.272-1.051)	0.070	0.609(0.301-1.233)	0.168	0.633(0.310-1.292)	0.209
Tertile 3	0.259(0.106-0.635)	**0.003**	0.250(0.094-0.659)	**0.005**	0.272(0.102-0.726)	**0.009**
**P for trend**		**0.007**		**0.018**		**0.031**

Model 1: was adjusted for age, gender, BMI, MAP + clinic factors (UA, eGFR and U-pro)+treatment . Model 2: was adjusted for Model 1 +Oxford classification(MEST-C). CI, confidence intervals; HR, hazard ratios. Bold values was that the differences were significant. BMI, Body mass index; MAP, mean arterial pressure; eGFR, estimated glomerular filtrationrate. U-pro, urine protein; UA, uric acid.

To reduce the influence of other related factors, we built two models that incorporated relevant clinical and pathological data. As shown in Models 1 and 2, multivariate Cox regression analysis indicated that HALP was an independent risk factor for renal progression even after adjustment for clinical parameters (age, sex, BMI, MAP, proteinuria, GFR, UA and treatment) (adjusted HR: 0.965, 95% CI: 0.943-0.988, P = 0.003) and combined with pathologic lesions (Oxford MEST-C) (adjusted HR: 0.967, 95% CI: 0.945-0.990, P = 0.006). However, when HALP was treated as a categorical variable, the effect of HALP on renal prognosis decreased significantly (as shown in **Table 2**).

**Figure 3:**
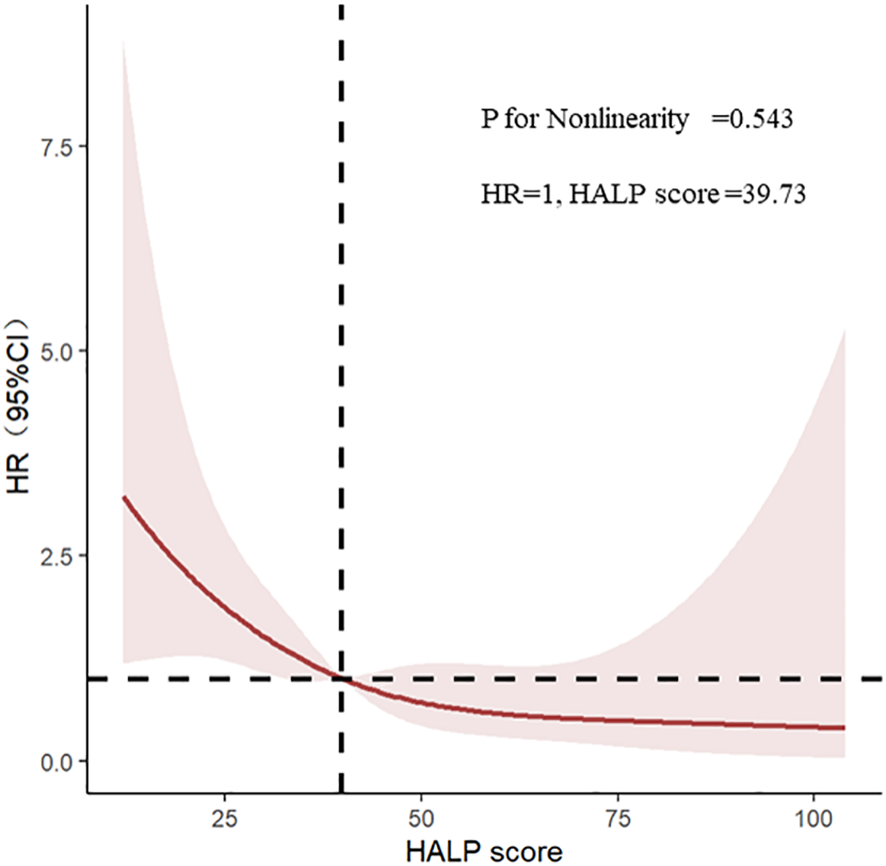
Restricted cubic splines of haemoglobin, albumin, lymphocyte, and platelet (HALP) and renal outcomes.

Through the multivariate adjusted restricted cubic splines, we found out that that there was a linear correlation between HALP and poor renal prognosis (P for nonlinearity = 0.543), showing a trend that the lower HALP was, the higher the risk of progression into ESRD (**Figure 3**).

### Subgroup analysis

**Figure 4:**
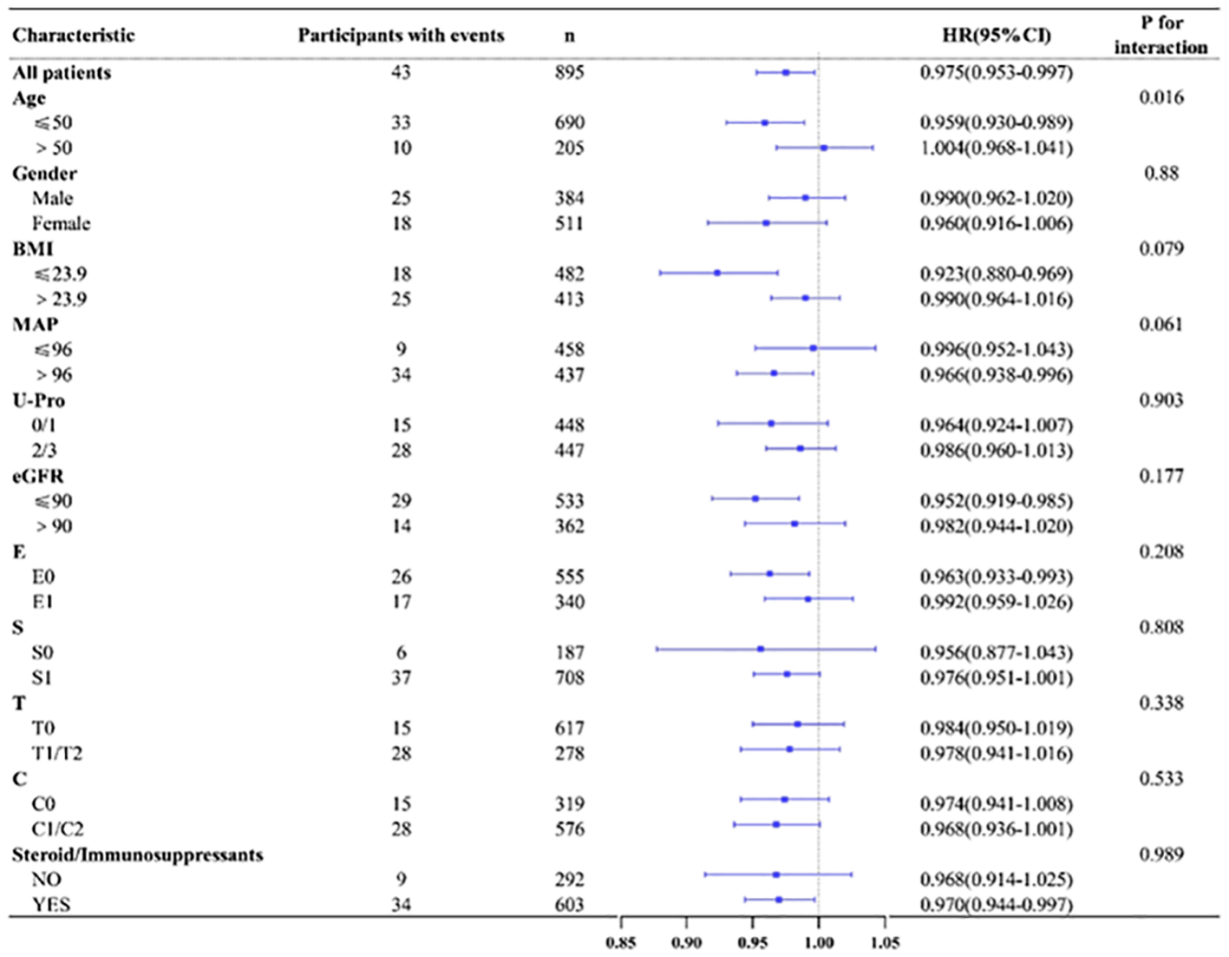
Forest plot of subgroup and interaction effects analyses.

According to age(≤50/>50), gender(male/female), BMI(≤23.9/>23.9),MAP(≤96 mmHg/>96 mmHg), U-pro(0-1/2-3), eGFR(≤90 mL/min/1.73 m2/>90 mL/min/1.73 m2) and renal pathological Oxford classification of IgAN (E0/E1,S0/S1,T0/T1-2,C0/C1-2), we made various subgroup analysis to evaluate any potential heterogeneity between different group. The results showed that HALP tended to have great predictive power in patients younger than 50 years old, with a BMI ≤ 23.9, eGFR ≤ 90 mL/min/1.73 m2 and who underwent steroid and/or immunosuppressant treatment (shown in **Figure 4**).

### Cut-off value for the ROC curve

**Figure 5:**
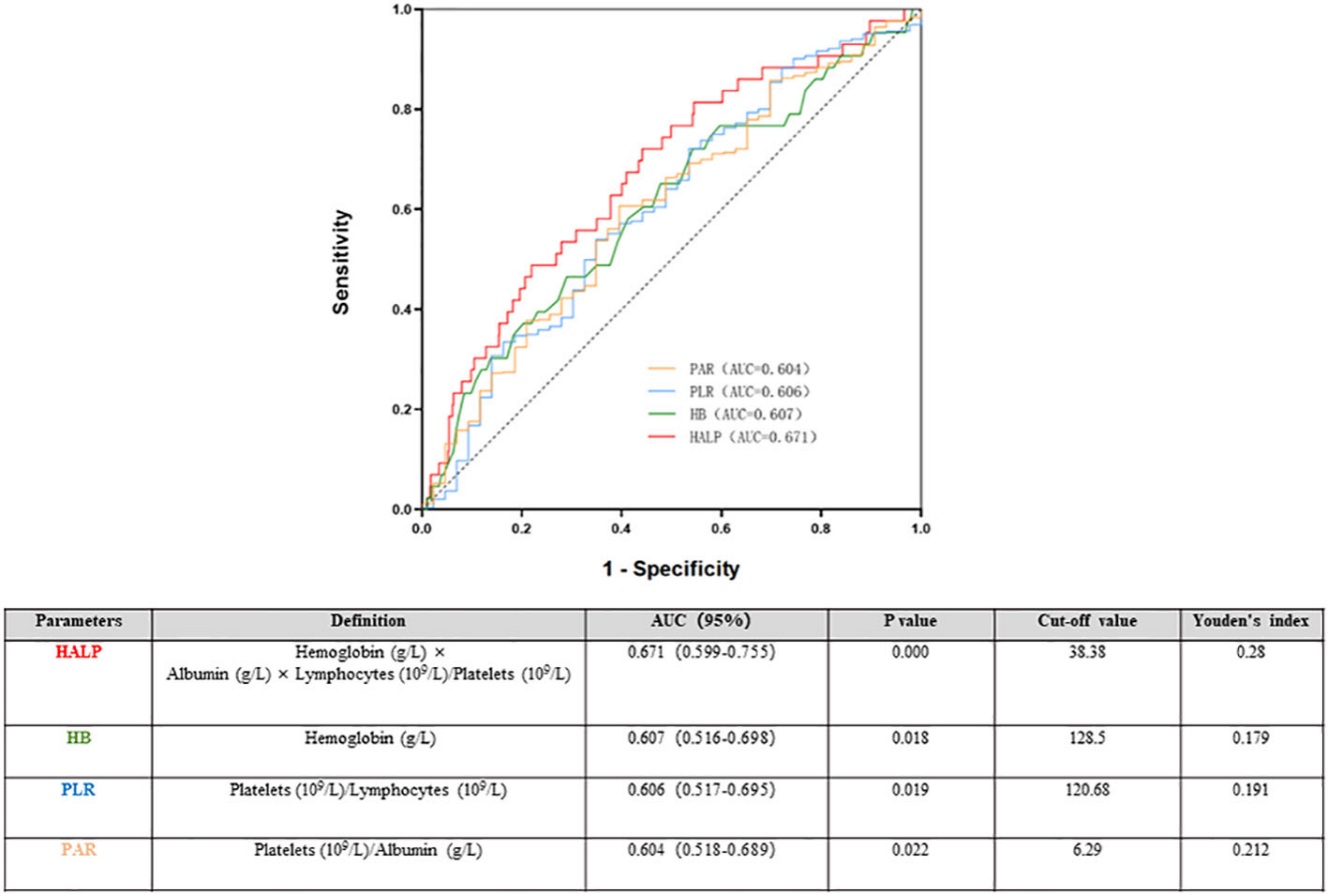
Time-dependent receiver operating characteristic (ROC) of haemoglobin, albumin, lymphocyte, and platelet (HALP) and renal outcomes.

The ROC was used to determine the predictive value of HALP. Compared with that of the routine inflammatory and nutrient factors Hb, PAR, and PLR, HALP had the highest AUC (0.662). The AUCs for Hb, PLR and PAR were 0.607, 0.604 and 0.606, respectively, which were lower than those of HALP. The results indicated that HALP has a higher predictive value for renal outcomes in IgAN, and the cut-off value of HALP in IgAN patients was 38.38, with a sensitivity of 72.1% and a specificity of 55.9% (**Figure 5**).

### The relationship between HALP score and renal prognosis in the matched cohort

Then, according to the cut-off value of HALP, all people were distributed into two group. high group (HALP ≥38.38) and low group (HALP <38.38). To eliminate the differences in clinical and pathological indices between the two groups, 1:1 propensity score(PS) matching using the greedy matching algorithm obtained matched pairs of 317 patients with low HALP and 317 patients with high HALP. As shown in **Table 3**, in the propensity score-matched cohort, there were no statistically significant differences in any of the covariates at baseline between the high and low groups.

**Table 3:** Baseline characteristics of the full cohort of 895 patients with IgAN according to **Cut-off value of HALP** and the propensity score matched cohort.

	Total	Before matching			After matching		
Variable		Low HALP group (≤38.38)	High HALP group (>38.38)	P value	Low HALP group (≤38.38)	High HALP group (>38.38)	P value
Participants, n	895	407	488		317	317	
Age, years	40.97±12.261	41.55±11.932	40.48±12.52	0.195	41.68±11.969	40.7±12.297	0.309
BMI, kg/m^2^	23.58±3.595	23.24±3.495	23.87±3.656	**0.010**	23.52±3.516	23.29±3.64	0.647
Male, n (%)	384 (42.9)	129 (31.7)	255 (52.3)	**0.000**	120 (37.9)	113 (35.6)	0.564
MAP, mmHg	96.13±14.055	96.48±14.312	95.84±13.844	0.494	95.78±13.832	94.88±14.235	0.420
U-pro, n (%)				**0.001**			0.462
-	96 (10.7)	34 (8.4)	62 (12.7)		31 (9.8)	30 (9.5)	
+	352 (39.3)	149 (36.6)	203 (41.6)		115 (36.3)	126 (39.7)	
++	367 (41.0)	173 (42.5)	194 (39.8)		136 (42.9)	137 (43.2)	
+++	80 (8.9)	51 (12.5)	29 (5.9)		35 (11.0)	24 (7.6)	
eGFR, ml/min/1.73m^2^	81.83±32.398	76.39±35.03	86.37±29.299	**0.000**	79.62±34.859	81.52±28.789	0.457
Uric acid, μmol/L	377.7±109.22	371.98±109.405	382.47±108.948	0.153	375.65±112.388	368.4±110.96	0.414
Mesangial cellularity, n (%)				0.183			0.499
MO	5 (0.6)	4 (1.0)	1 (0.2)		2 (0.6)	0 (0)	
M1	890 (99.4)	403 (99.0)	487 (99.8)		315 (99.4)	317 (100)	
Endocapillary hypercellularity, n (%)				**0.011**			0.628
E0	555 (62.0)	234 (57.5)	321 (65.8)		190 (59.9)	184 (58.0)	
E1	340 (38.0)	173 (42.5)	167 (34.2)		127 (40.1)	133 (42.0)	
Segmental sclerosis, n (%)				0.097			0.920
S0	187 (20.9)	75 (18.4)	112 (23.0)		60 (18.9)	61 (19.2)	
S1	708 (79.1)	332 (81.6)	376 (77.0)		257 (81.1)	256 (80.8)	
Tubular atrophy/interstitial fibrosis, n (%)				**0.000**			0.494
T0	617 (68.9)	252 (61.9)	365 (74.8)		213 (67.2)	221 (69.7)	
T1+T2	278 (31.1)	155 (38.1)	123 (25.2)		104 (32.8)	96 (30.3)	
Crescents, n (%)				**0.024**			0.800
C0	319 (35.6)	129 (31.7)	190 (38.9)		107 (33.8)	104 (32.8)	
C1+C2	576 (64.4)	278 (68.3)	298 (61.1)		210 (66.2)	213 (67.2)	
Therapy, n (%)				**0.000**			0.070
None Steroid	292 (32.6)	100 (24.6)	192 (39.3)		80 (25.2)	106 (33.4)	
Steroid alone	191 (21.3)	91 (22.4)	100 (20.5)		75 (23.7)	63 (19.9)	
Steroid+Immunosuppressants	412 (46.0)	216 (53.1)	196 (40.2)		162 (51.1)	148 (46.7)	

BMI, Body mass index; MAP, mean arterial pressure; eGFR, estimated glomerular filtration rate ;Continuous variables are expressed as mean±standard deviation or as median (interquartile range). Categorical variables are expressed as frequency (%). Bold values was that the differences were significant.

**Figure 6:**
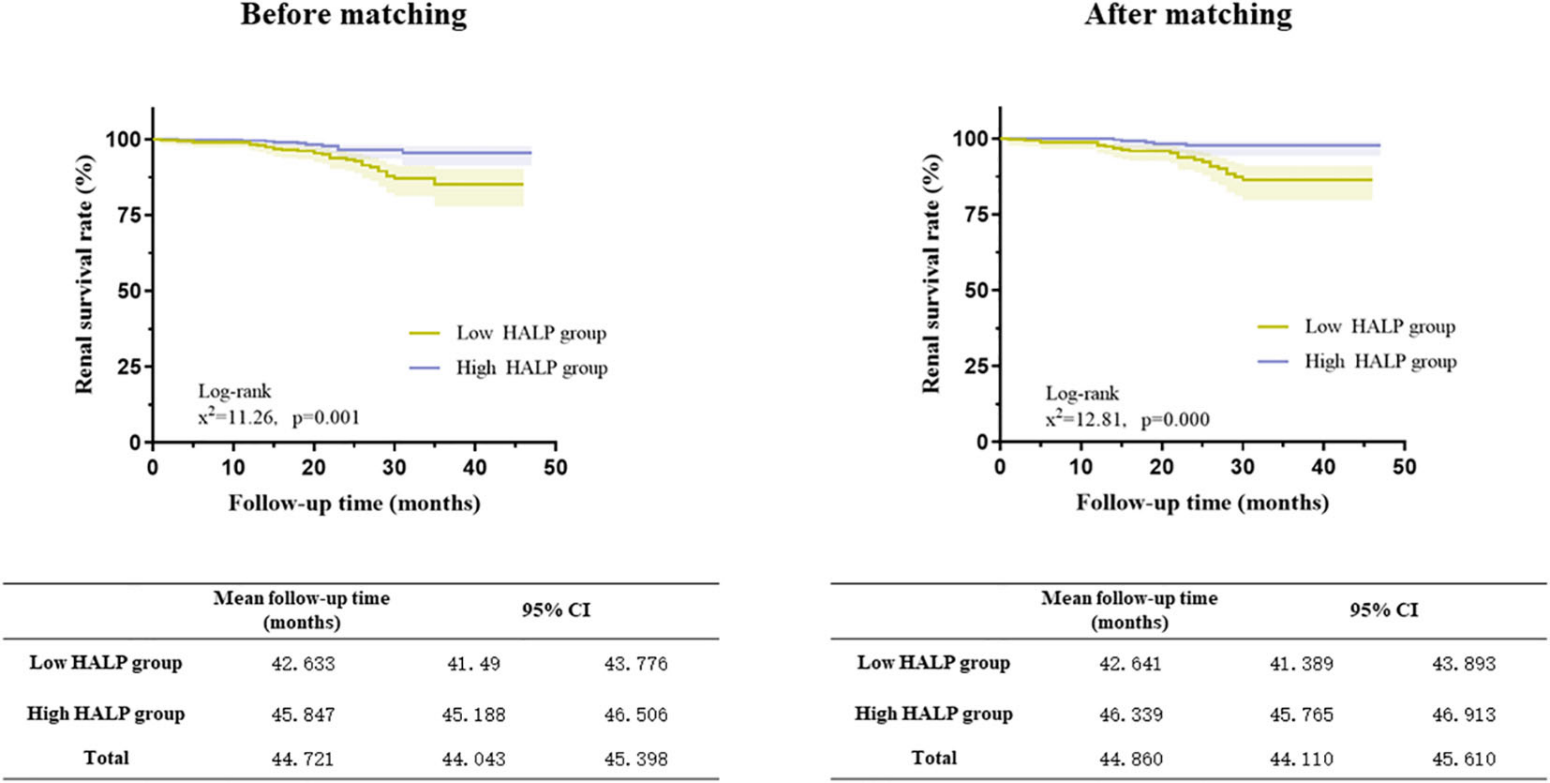
Relationship between HALP score and renal outcomes in the full cohort and the propensity score-matched cohort (Kaplan–Meier curves).

Kaplan−Meier analyses showed that patients in the low HALP group had a higher risk of developing kidney failure than those in the high HALP group in the matched cohort (log-rank =12.81, p = 0.001, shown in **Figure 6**). In the high group, the mean renal survival time was much longer than that in the short group.

After PS, the unadjusted analysis showed that a lower level of HALP (continuous or categorical variable) was associated with an increased risk of developing kidney failure (HR =0.966 (CI: 0.946-0.987) and HR =0.338 (CI: 0.173-0.657), respectively). After adjusting for clinicopathological parameters, HALP remained an independent risk factor for renal prognosis. (HR 0.222, CI: 0.084-0.588, P=0.002) (**Table 4**).

**Table 4:** Relationship between HALP score and renal outcomes in the full cohort and the propensity score-matched cohort (Multivariate Cox regression analysis).

	Before matching					After matching				
	Continuous	P	Low HALP (≤38.38)	High HALP (>38.38)	P	Continuous	P	Low HALP (≤38.38)	High HALP (>38.38)	P
**Number of participants with events/n**	43/895		31/407	12/488		30/634		25/317	5/317	
**Crude Model**	0.966 (0.946-0.987)	**0.001**	1.00 (reference)	0.338 (0.173-0.657)	**0.001**	0.952 (0.925-0.979)	**0.001**	1.00 (reference)	0.206 (0.079-0.537)	**0.001**
**Model 1**	0.965 (0.943-0.988)	**0.003**	1.00 (reference)	0.347 (0.168-0.714)	**0.004**	0.953 (0.924-0.983)	**0.003**	1.00 (reference)	0.229 (0.087-0.604)	**0.003**
**Model 2**	0.967 (0.945-0.990)	**0.006**	1.00 (reference)	0.368 (0.178-0.760)	**0.007**	0.951 (0.922-0.982)	**0.002**	1.00 (reference))	0.222 (0.084-0.588)	**0.002**

Model 1: was adjusted for age, gender, BMI, MAP + clinic factors (uric acid, eGFR and U-pro)+treatment. Model 2: was adjusted for Model 1 +Oxford classification(MEST-C). CI, confidence intervals; HR, hazard ratios. Bold values was that the differences were significant.

These results indicated that baseline HALP is an important indicator of renal prognosis, and the probability of poor renal prognosis in patients with HALP scores less than 38.38 was much higher than that in patients with HALP scores ≥38.38. Baseline HALP can effectively assess renal outcomes.

### The Assessments of the IIgAN-PRT model with renal interstitial inflammation

**Figure 7:**
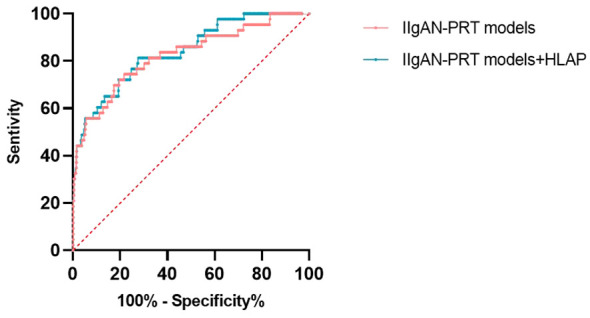
The ROC of IIgAN-PRT models with and without HALP.

We made muti logistic regression with the relevant parameters in the original IIgAN-PRT models with and without HALP. The ROC indicated a higher AUC for the model with HALP(AUC: 0.841) compared with that without HALP(AUC: 0.824),as shown in **Figure 7**.

## Discussion

IgAN is the most common form of primary glomerulonephritis (14). Many factors are related to the development and progression of IgAN, and early diagnosis and accurate prognosis assessment are crucial for preventing IgAN from progressing to ESRD. As a kind of autoimmune disease, various studies have found that the infiltration of inflammatory cells, the secretion of inflammatory factors, and the activation of the inflammatory response play important roles in IgAN.

The HALP score was first introduced by Chen et al. calculated based on haemoglobin, albumin, lymphocyte and platelet counts, reflecting the patient’s nutritional and immune status (9). Serum albumin was widely used as a reliable biomarker for systemic inflammation and nutritional status. It is also an acute-phase protein that responds to systemic inflammation (15). Albumin-based combined indices, such as the albumin-to-fibrinogen ratio (AFR) and uric acid-to-albumin ratio (UAR), have been reported as independent predictors of corticosteroid response and prognosis for IgAN (16–18). Low serum albumin may result in oxidative stress damage and inflammation due to the effects of cytokines(IL-6,TNF-α) (19). Lymphocytes, consisting of differential subpopulations, usually reflect the nutrition and immune state. A decrease of lymphocytes is a hallmark of inflammatory disease progression. Previous studies have demonstrated that lymphocyte based the combination indicator (neutrophil-to-lymphocyte ratio) is an independent marker for poor renal prognosis in IgAN and adult IgA vasculitis (20, 21). Emerging evidence has shown that platelets are positively related to some chronic inflammatory markers, as an novel prognostic indicators in inflammatory diseases (22–24). Through interacting with immune cells and secreting various proinflammatory cytokines, platelets participate in triggering and aggravating inflammation (25). Tan once reported that platelets (PLT), Platelets/Lymphocytes (PLR), and Platelets/Albumin (PAR)were correlated with the renal outcomes in IgAN, and PAR was an independent risk factor (26). aemoglobin is an important indicator of anaemia. Many studies have indicated that anaemia results in a reduced oxygen-carrying capacity through depriving the kidneys of needed oxygen, to promoted the progression of kidney disease (27). In a retrospective study of 619 patients with IgAN, Xie et al. (28) reported that haemoglobin was one of the predictor factors for developing into ESRD.Previously, our centre also identified that a low level of haemoglobin was nonlinearly associated with IgAN progression. Anaemic IgAN patients presented a higher risk of developing poor outcomes than non-anaemic patients (29). As a novel comprehensive marker, HALP has been reported to reflect inflammation and nutritional status and found to be closely related to the prognosis of various tumours (7–10). Zhang et al. recently reported HALP as an indicator related to cardiovascular and all-cause mortality in general and haemodialysis patients (11, 30), while as far as I know, there have been limited studies on the correlation between HALP and IgAN prognosis. In this retrospective study, we demonstrated that a lower HALP score in IgAN patients was associated with more severe clinical features and pathologic lesions. Comparison of endpoint events showed that the incidence of endpoint events in the HALP 1 group was significantly higher than that in the other groups. K−M survival curves showed significant differences in the renal survival rate among three groups. In the lower HALP group, the mean kidney survival time was much shorter than that in the other groups. Multivariate Cox regression further indicated that HALP was an independent risk factor for renal prognosis. At the same time, we compared the prediction value of the IIgANPRT model with and without HALP, The result showed that the assessments of the IIgANPRT model was also improved by adding HALP. These suggest that HALP may be a predictor for kidney function progression in IgAN.

As we know, Many clinical indicators affect each other and there are many confounding factors, such as the value of serum albumin could be affected by proteinuria, to eliminate various affects, more accurately evaluate the predictive power of HALP, through subgroup analysis we further analysed the predictive effect of HALP with different clinicopathological in IgAN. These results seemed to show that HALP has stronger predictive value when patients already have renal impairment and undergo steroid and/or immunosuppressant treatment. In younger and lower BMI patients, baseline HALP assessment was more valuable. These results confirmed that the value of HALP in the evaluation of renal prognosis is not only due to the effect on renal function.

Various studies have identified the prognostic value of PLT-based markers, such as PLR and PAR (26). Compared with traditional parameters, HALP has the best predictive efficiency, with an AUC of 0.671. According to the HALP cut-off of 38.83, all patients were then divided into two groups. The results were similar to those of a previous study; the low HALP group had serious clinical and pathological lesions. To reduce the influence of other parameters, we performed propensity score matching among all participants. The results show that lower HALP was associated with an increasing risk of poor renal outcome in IgAN both in the full cohort and the matched cohort. After adjusting for clinicopathological parameters, HALP remained an independent risk factor for renal prognosis in the matched cohort.

Previously, we believed that HALP was correlated with the prognosis of IgAN, mainly because patients with lower HALP had more serious clinical and pathological manifestations. However, after excluding the influence of related factors through propensity matching and multivariate Cox regression analysis, HALP remained an important prognostic factor. We thought that HALP mainly reflects the inflammatory state of the patients to indicate the prognosis of IgAN.

Emerging studies have suggested that the systemic inflammatory response and nutrition status play vital roles in the progression of IgAN (20, 26). As an autoimmune disease, the infiltration of inflammatory cells, secretion of inflammatory factors, and oxidative stress damage play important roles in IgAN. Groza reviewed the role of IL-6 and its related pathways in the development of IgAN (31). Xie found that the intensity of macrophage infiltration in glomerular predicted the response to immunosuppressive therapy in IgAN especially when patients with higher risk of progression (32). Our centre also reported that degree of interstitial inflammation is another important pathologic parameter that can be used as a predictor for kidney disease progression in IgAN (33). Although these indicators have been indicated to be associated with IgAN prognosis and therapeutic response, they require additional examination or evaluation by a senior pathologist and are difficult to popularize. In this study, we found that the HALP acted as a valuable and composite index that reflects the status of nutrition and inflammation, which is easy to obtain through routine clinical blood tests and is much cheaper than other inflammatory indicators and pathology examinations and is expected to be a new marker indicating the prognosis of IgAN.

Some limitations warrant consideration. First, this study was a single-centre retrospective study with an insufficient sample size and lack of information in non-Chinese population,in the future we look forward to expanding the sample and conducting multicentre studies, and used public databases to conduct different ethnically specific studies. Second, the duration of follow-up was relatively short. As a chronic disease, the short follow-up time in IgAN may result in some endpoint events not being detected, affecting the predictive efficacy of HALP. Therefore, we will further extend the follow-up time, to clarify the significance and value of HALP in in evaluating long-term prognosis of IgAN patients. Finally, more studies on the effects of HALP and glucocorticoid treatment response and the short-term and long-term prognosis of IgAN need to be further refined.

## Conclusion

HALP is a significant and independent risk factor for renal disease progression in patients with IgAN, especially when patients are younger, have lower BIM, impaired kidney function or need steroid and/or immunosuppressant treatment. Patients with a HALP score of less than 38.38 at renal biopsy need more attention and more active intervention.

## Data availability statement

The original contributions presented in the study are included in the article/supplementary material. Further inquiries can be directed to the corresponding author.

## Ethics statement

The studies involving humans were approved by Hangzhou TCM Hospital Affiliated to Zhejiang Chinese Medical University (2023KLL001). The studies were conducted in accordance with the local legislation and institutional requirements. Written informed consent for participation was not required from the participants or the participants’ legal guardians/next of kin in accordance with the national legislation and institutional requirements.

## Author contributions

YY: Writing – original draft. XL: Formal analysis, Methodology, Writing – review & editing. MH: Data curation, Writing – review & editing. YW: Data curation, Writing – review & editing. XJ: Funding acquisition, Writing – review & editing.
